# Challenges to obtaining parental permission for child participation in a school-based waterpipe tobacco smoking prevention intervention in Qatar

**DOI:** 10.1186/1472-6939-15-70

**Published:** 2014-09-30

**Authors:** Rima T Nakkash, Ahmad Al Mulla, Lena Torossian, Roubina Karhily, Lama Shuayb, Ziyad R Mahfoud, Ibrahim Janahi, Al Anoud Al Ansari, Rema A Afifi

**Affiliations:** Faculty of Health Sciences, American University of Beirut, PO Box 11–0237, Riad El Solh, Beirut, 1107 2020 Lebanon; Smoking Cessation Clinic, Medicine Department, Hamad Medical Corporation, P.O. Box 3050, Doha, Qatar; Medicine Department, Hamad Medical Corporation, P.O. Box 3050, Doha, Qatar; Weill Cornell Medical College in Qatar, Doha, Qatar; Smoking Cessation Clinic, Medicine Department, Hamad Medical Corporation, P.O. Box 3050, Doha, Qatar

**Keywords:** Informed consent, Parental consent, Qatar, School, Intervention, Waterpipe

## Abstract

**Background:**

Involving children in research studies requires obtaining parental permission. A school-based intervention to delay/prevent waterpipe use for 7th and 8th graders in Qatar was developed, and parental permission requested. Fifty three percent (2308/4314) of the parents returned permission forms; of those 19.5% of the total (840/4314) granted permission. This paper describes the challenges to obtaining parental permission. No research to date has described such challenges in the Arab world.

**Methods:**

A random sample of 40 schools in Doha, Qatar was selected for inclusion in the original intervention. Permission forms were distributed to parents for approval of their child’s participation. The permission forms requested that parents indicate their reasons for non-permission if they declined. These were categorized into themes. In order to understand reasons for non-permission, interviews with parents were conducted. Phone numbers of parents were requested from the school administration; 12 of the 40 schools (30%) agreed to provide the contact information. A random sample of 28 parents from 12 schools was interviewed to reach data saturation. Thematic analysis was used to analyze their responses.

**Results:**

Reasons for non-permission documented in both the forms and interviews included: poor timing; lack of interest; the child not wanting to participate; and the child living in a smoke-free environment. Interviews provided information on important topics to include in the consent forms, parents’ decision-making processes regarding their child’s participation, and considerations for communicating with parents. Many parents also indicated that this was the first time they had been asked to give an informed consent for their child’s participation in a study.

**Conclusions:**

Results indicate that more attention needs to be given to the informed parental consent process. Researchers should consider enhancing both the methods of communicating information as well the specific information provided. Before embarking on recruitment of children for studies, formative research on the parental consent process is suggested.

**Electronic supplementary material:**

The online version of this article (doi:10.1186/1472-6939-15-70) contains supplementary material, which is available to authorized users.

## Background

A variety of procedures have been suggested to enhance the protection of human participants in research [1–3]. One key process is informed consent, which requires obtaining participants’ agreement to participate in a research study after fully informing them about the study’s content and requirements [3, 4]. Informed consent entails describing the study to participants, stating the risks and benefits, stressing the voluntary nature of participating in the research, ensuring through research design that no coercion is present, and informing participants of how to contact the research team and the institutional review board office (if needed) [2, 3, 5–7]. In gathering all the necessary information for an informed consent, several population groups that are considered to be vulnerable are afforded special consideration. Involving children in research studies, for example, requires obtaining parental permission prior to approaching them for their assent.

High participation rates in research ensure representativeness of the sample to the larger population and minimize bias in interpretation of results. Many studies involving children have documented challenges to parental permission, and recommend oversampling to achieve an adequate sample size [4, 8]. A recent review of 500 school-based projects found that only 11.5% reported on procedures to gain parental permission and on participation rates [9]. Strategies to increase parental permission have included tips for the content of the permission form itself [4, 8, 10–12]; incentivizing permission for parents, student, and teachers [13–15]; using participatory methods; and active communication techniques [14, 16–18]. Attention to these issues have been found to be particularly important in research studies with complex designs such as Randomized Controlled Trials (RCTs) as the concept of randomization is difficult to understand, making many parents reluctant to give their consent [12, 19, 20]. Type of consent (active versus passive) has also been found to influence response rates. Passive consent, the requirement for parent to actively decline their child’s participation, tends to enhance participation rates. When active consent (the requirement to actively accept child participation) is used, strategies should be put in place to enhance participation [21]. Other important factors to explore include perceived relevance and acceptance of the research by and commitment of administrators, e.g., provision of class release time for students and program facilitators [18]. Generally, the literature suggests tailoring different recruitment strategies for different subpopulations [21].

The importance of cultural considerations in consent taking has also been noted [5, 12, 22–25]. Despite this, research into the application of ethical principles for human participant protection with children is limited in the Arab World. The Arab world ranks lowest globally in research productivity and output [26–28], which suggests that issues surrounding parental consent might be significantly different than in other settings, and therefore require different strategies.

This paper reviews challenges to obtaining parental permission for students’ participation in a school-based tobacco control intervention in Qatar. Qatar is a small country (4,416 square miles) in the Arabian Gulf, bordering Saudi Arabia to the south, with the rest of its territory surrounded by the Persian Gulf. According to the Qatari Statistics Authority, the 2010 population size is estimated to be 1,853,563 with 3/4 of its population being male [29]. This disproportionate gender distribution is due to the size of the foreign worker population. Only 20% of the population is Qatari nationals; the rest are other Arabs, Indians, Filipinos, Nepalese, Pakistanis, and Sri Lankans who are guest workers in Qatar [29]. The Qatar National Research Foundation is investing heavily in research. However the research environment is nascent [30]. In recent years, a number of US universities have established campuses in Doha, and their faculty are held to the same academic standards as those in the US, where expectations are to conduct and publish research.

There has been a recent increase in the use of waterpipe tobacco by young people worldwide, including the Arab world [31, 32]. According to the Global Youth Tobacco Survey, 25.2% of males and 13.1% of females aged 13–15 used any tobacco product in Qatar, including waterpipe smoking [33]. Use of waterpipe was more prevalent than cigarette smoking among this age group in Qatar [34]. In order to prevent or delay initiation of waterpipe use, a school-based intervention study was developed for 7th and 8th graders in Qatar. The intervention was part of a larger study that also included Lebanon. The intervention was theory-based and consisted of 8 skill-building sessions conducted during school time. The evaluation design was a pre-test post-test randomized control trial. The pre and post assessments included a survey as well as saliva testing on a randomly selected sample of students to measure cotinine levels.

As participants (in 7th and 8th grades) were less than 18 years of age, parental permission was required before the students could participate. About 50% parents returned the permission form and about 20% gave permission for their child’s participation, despite the fact that the content of the permission form followed suggested best practices in the literature, including active communication with principals and students about the permission forms. However, the use of incentives, a best practice for improving participation when using active consent, was neither feasible nor acceptable in our context.

This paper describes challenges to obtaining parental permission for the waterpipe tobacco smoking prevention study. We begin by describing the process and outcomes of obtaining parental permission for their child’s participation in this research, and then describe reasons given by parents for their choice. To do that, we quantitatively analyzed written responses of parents to a question on the consent form regarding reasons for non-consent; and qualitatively analyzed follow-up phone interviews with parents who gave permission or did not. Individual interviews provide an opportunity to understand people’s experiences in more depth without the biases of group social norms, and therefore were considered to be the most effective way to further understand the challenges to parental permission [35]. Results of this research can inform the international literature on the process of parental permission for school-based interventions, and add to the literature on this topic in the Arab world, and more specifically in Qatar.

## Methods

### Study setting

Public and private schools in Doha, Qatar served as the setting for this study. The Supreme Council of Education is responsible for managing the educational system in Qatar. Different schools are available to support both national and expatriate population [36]. All Qataris can attend public schools free of charge. Non-Qatari expatriates have the option of choosing between public and private schools. Private international schools are sponsored by country embassies and are a type of private school that offers curriculum from a country other than the one of Qatar. These schools are generally attended by children of expatriates of those countries (e.g., Indians, British, Lebanese, Pakistani, Americans) although some Qatari nationals choose to send their children to these schools.

### Sources of data

In order to understand the reasons for the low permission rate, two primary sources of data were used: (1) parents’ feedback on the permission forms (quantitative methodology), and (2) individual phone interviews with parents (qualitative methodology). Each is described separately below.

### Parent feedback on permission forms

#### Study selection and recruitment

A random selection of 40 schools located in the capital city of Doha were included in the original sample for the intervention. Students in grades 7 and 8 were included. Subsequently, the schools were randomly assigned to 2 groups: control (20 schools) and intervention (20 schools). The 40 schools included Qatari schools as well as Moroccan, Egyptian, Indian, Pakistani, Lebanese, American and British schools. The parental permission forms were shared with and approved by the principals of the schools.

#### Data collection

The forms were then distributed by the research team to the students in class who were asked to give them to their parents and return them the next day. The research team explained to the students the purpose of the intervention study and the permission forms. In addition, the majority of the schools sent text messages by phone to all parents on the same day informing them of the need to read and send the forms back to school. Schools that did not have the text message system sent circulars home along with the permission forms.

The permission form was modeled on best practices in the literature as described above. It included: information about the purpose of the study, the details of what participation entailed, the duration of the study, the risks and benefits, the confidentiality of information obtained about participants, their rights, and the contact information of the principal investigator and the Institutional Review Board (IRB). Parents were asked to indicate on the form whether or not they grant permission for their child’s participation by checking the appropriate box, i.e., in both cases the form needed to be returned. Moreover, the form asked parents who indicated that they did not give permission to write in their own words the reasons behind this decision. Despite the literature indicating that passive consent enhances participation rates, active consent was used in this research for the following reasons: (i) in a nascent research environment, active consent was felt to enhance awareness of research processes; (ii) in a socio-cultural context where it is expected that parents govern decisions regarding their children, active consent was believed to protect parental rights and be more acceptable; and (iii) the consent form also requested permission to take a saliva sample which is a more invasive procedure that was deemed to require active consent.

Since few forms were returned the following day, the time for return of forms was extended to one week. A second set of forms was then sent to parents who had not yet returned them. In total, of the 4314 parents to whom permission forms were sent, 2308 (53.5%) returned them. Of those, 840 (about 20% of the total) agreed to their child’s participation in the study while 1468 refused.

#### Data analysis

The status of permission for each child from the 40 schools was entered into SPSS (version 18) as were the reasons given for non-permission. These reasons were summarized and grouped into themes. Not all parents provided a reason. All who did respond provided only one reason, and the responses were generally short.

### Individual phone interviews with parents

#### Study selection and recruitment

The list of phone numbers of parents from schools was requested from the school administration; 12/40 schools (30%) agreed to provide the contact information. Table 1 indicates that there were no significant differences between schools that agreed to provide this information and schools that did not on: gender of students in the school, percentage of returned permissions by parents and percentage of parents who accepted children’s participation in the study.

**Table 1:** Characteristics of schools who agreed to allow parents to be contacted for the qualitative interviews and schools who did not*

Variable	Schools that agreed to provide parental contact information	Schools that did not agree to provide parental contact information	P-value
**Schools with**	N** (%)	N** (%)	0.666
Only boys	6 (50.0%)	9 (32.1%)	
Only girls	4 (33.3%)	13 (46.4%)	
Co-educational	2 (16.7%)	6 (21.4%)	
**Mean (sd) percent of parents returning consent forms**	62.7% (29.1%)	48.2% (26.2%)	.130
**Mean (sd) percent of parents who accepted children’s participation**	24.7% (16.1%)	16.3% (12.0%)	.077

*The sample size may limit the possibility of meaningful statistical analysis of differences for this question.

**N = number of schools.

The approved IRB protocol proposed 25–30 interviews (and continuing until data saturation was reached). In order to ensure selection of parents from all schools, 3 parents from each of the 12 schools were randomly selected with the following considerations: equal representation of grades 7 and 8; of boys and girls; and of parents who had returned the permission forms, with or without granting permission.

Parents were contacted to schedule interviews; saturation was reached at 28 interviews. In order to reach these 28 parents who accepted to be interviewed, 200 were contacted and 56 reached. Parents included those who had originally consented and those who had not. Saturation was reached for both at 28 interviews. Interviews were conducted in English or Arabic, depending on the preference of the interviewee. The nationalities of the interviewees were Qatari, Indian and Pakistani. None of the refusals for participation in the interviews was due to language barriers.

#### Data collection

Phone interviews were conducted in June 2011 (about 5 months after consent forms were first distributed) and took between 15–30 minutes each to complete. All 3 female interviewers had a Master Degree in Public Health (LT, RK, LS). The interviewers - who were familiar with the research study and had participated in the recruitment process - initially contacted parents through phone calls, introduced themselves, and initiated the informed consent process which included giving a short introduction about the purpose of the call, and making clear that the information provided by the parents would not be shared with the school and would be analyzed with the exclusion of names and phone numbers. If the parent agreed to be interviewed, s/he was asked whether they preferred to continue the interview on the phone or in person. Interviews were recorded when approval was granted. However, in case parents refused recording, the interviewer indicated that a notetaker would be present to document parents’ responses.

The interview protocol included questions regarding: (1) the value of the information delivered in the permission form with respect to parents’ decision-making; (2) the modes of communication utilized by the school to inform parents about new programs; and (3) the methods researchers should use to inform parents about such programs. In addition, general demographic information (age, gender, level of education of husband and wife, and perception of income as compared to others) was requested from each interviewee. The specific questions were tailored to whether the parents had given permission, not given permission, or did not remember receiving any information. For the latter group, the interviewer briefly described the consent form before proceeding with the questions.

#### Data analysis

Consent decision for the 28 parents that were included in the interviews was abstracted from the consent form. Descriptive analysis compared the decision by parent gender and child gender.

Interviews were transcribed, coded, and analyzed using thematic analysis, a process of coding qualitative data according to themes or patterns identified in the transcripts. Coding is an essential step in the organization, processing and analysis of qualitative information as it lays the groundwork for the interpretation phase [37]. Analysis was conducted in Arabic for interviews conducted in Arabic to avoid loss of meaning resulting from translation. Responses to the interview questions of the parents who had originally given permission and those who had not were compared. There were no thematic differences in response to the questions among these two groups.

### Ethical review

Before the start of the intervention study, ethical approval was obtained from the Institutional Review Board (IRB) at Hamad Medical Center. Approval for the study was also obtained from the Supreme Council of Education in Qatar. Consent for school participation was obtained orally from principals of the participating schools. The research team submitted a detailed follow-up protocol and received IRB approval from Hamad Medical Center to conduct individual interviews with parents with the aim of understanding the consent process more thoroughly.

## Results

Results of the two forms of data collected will be presented separately.

### Parents’ feedback on permission forms

Out of 1468 parents who refused their child’s participation in the research, 540 (38%) wrote the reason for their choice (Table 2). Many parents (24.8%) stated that the timing during which the study was being conducted was inappropriate since it fell during school exam period. Other reasons given were lack of interest in the topic among parents (13.3%) or their child (10.9%). Parents also listed as a reason that the child lives in a smoke free environment and hence they saw participation as needless (9.8%).

**Table 2:** **Reasons given by parents for not consenting to their child’s participation in the study**

Reason for not consenting	N = 540 (%) as mentioned in consent form
1. Inappropriate timing for study implementation (exam period, etc.…)	24.8%
2. Parent not interested	13.3%
3. No specific reason given	12.2%
4. Child doesn’t want to participate	10.9%
5. Child lives in a smoke-free environment	9.8%
6. Child does not need to participate	8.5%
7. Personal reasons	5.3%
8. The topic is more relevant to boys than to girls	2.9%
9. Others (Afraid that the child might be harmed, Location of study implementation not clear, Objection to saliva collection, Responsibility of family to explain to the child about such topics)	3.1%
10. Opening the child’s eyes to topic s/he should not know about	2.0%
11. Child’s health	2.0%
12. Risks and benefits of study not clear	1.8%
13. The child is too young for the topic	1.7%
14. Parent doesn’t understand the topic or thinks doesn’t think s/he should participate	1.7%
**Total**	**100.0**%

### Interviews with parents

Of the 28 parents who agreed to an interview, about half were fathers (n = 13, 46.5%), over half were 41 years of age and older, and many (77.0% of fathers, 66.6% of mothers) had completed secondary level education or above. In response to the question about socioeconomic status of the family, the majority (78.5%) indicated that they consider themselves to have the same income as parents of other children who attend their child’s school. About half (n = 13) of the interviewees were parents of girls.

The consent decision for these parents, analyzed by gender of child and gender of the parents, suggests that fathers were slightly more likely to give permission for their child’s participation than mothers, and that parents of either gender were slightly more likely to give permission to daughter’s participation than son’s (Table 3).

**Table 3:** **Analysis of parental consent decision by child and parent gender for the 28 parents who were interviewed**

	Parent consented		
	Yes	No	% Yes
**Gender of child**			
Male	7	6	53.8%
Female	9	6	64.3%
**Gender of parent**			
Male	8	5	61.5%
Female*	8	7	53.3%

*one of the female respondents was an older sister rather than a mother.

When asked about their decision regarding their child’s participation, the majority remembered their decision (11/16 (69%) of those who consented and 9/12 (75%) of those who did not). Responses by parents in the interviews were categorized into three main themes: (i) information on the locus of decision making for participation of children in activities; and suggestions they had for (ii) the consent form itself, and (iii) methods of communicating with parents. Additional file 1: Table S4 identifies these main themes and includes examples of quotes.

### Locus of decision making and participation

Parents reported that decision making around their child’s participation in any program happens in one of three ways: i) the parent makes the decision first and if s/he approves participation, asks their child is they are interested in participating, ii) the child is asked whether or not s/he would like to participate and the parents then make the final decision, or iii) all of the family sits and discusses the issue and makes the decision together. The first was the most common process for decision-making among this group of parents. Almost all parents stated that their children had not participated in any research studies before and this is the first instance that they have been asked to give permission for their child’s participation in a research study.

### Content of the informed permission form

With respect to the information in the permission form, most parents thought that the information was sufficient and clear, particularly pertaining to the detailed description of the study. Some parents also expressed the particular importance to them of the information provided in the forms regarding confidentiality and voluntariness. Some of the interviewees made suggestions to include more information about (i) the details of the intervention sessions such as the topics of each session, (ii) the mode of session delivery, and (iii) the location and timing of these sessions.

When asked about the reasons for giving or refusing permission, the parents who had approved their child’s participation viewed the topic being discussed as important and beneficial for their child. On the other hand, the reasons given by the parents who refused their child’s participation were similar to those stated on the permission forms (summarized in Table 2): parents’ lack of interest, inappropriate timing for study implementation, child’s lack of interest, child lives in smoke free environment and hence the perception that this intervention is irrelevant. In addition, interviewed parents mentioned that this topic is more relevant to boys than girls (due to their perceptions about the risk of smoking initiation).

As mentioned previously, the permission form also noted that a sample of saliva would be taken from a random number of students. Researchers had thought this may be an obstacle to giving permission. However, only two the interviewed parents mentioned this as a reason for not giving permission.

### Communication with parents

The majority of interviewed parents mentioned that the school usually communicates with them through written documents and/or mobile text messages. However, for the purpose of information on research studies, they preferred direct communication, either through phone calls or face-to-face meetings conducted at times that were convenient to them.

## Discussion

This study outlines challenges to obtaining parental permission in a school-based waterpipe tobacco control prevention intervention in Qatar, and reasons parents gave for their choice to give permission or not. Findings were obtained from two data sources, the permission forms where parents stated the reasons for non-permission and the follow up qualitative individual interviews with parents. These sources were triangulated and served to build our understanding of challenges to parental permission. Indeed, the answers on the permission forms were validated by the findings from interviews. These centered on concerns that participation might compete with academic priorities as well as perceptions that interventions such as these are either not needed as the child already lives in a non-smoking environment and knows all about tobacco; or that interventions like these raise attention unnecessarily to a risky behavior that is better not discussed. None of these specific issues of content has been raised in the literature before. Both of these perceptions are common in health education but both are faulty. Though parental smoking is a risk factor for tobacco use, many young people whose parents do not smoke also try out and continue smoking. This is especially true with the waterpipe as evidenced by the fact that rates of smoking among young people outweigh prevalence rates in adults [38]. With respect to the latter concern about exposure, evidence has shown that mere exposure to a topic does not enhance probability of engagement in it, but rather protects against risky behavior [39]. These concerns however, point to the importance of addressing these specific misperceptions prior to initiating an intervention or seeking parental consent.

Findings did not indicate, as other studies have, that parents were unable to understand or read the consent; parents in this study indicated that they understood the information provided in the consent form and thought it was sufficient [8]. This may be due to the selective sample we accessed (described further below). However, their responses do suggest the need for more detail on the specifics of the intervention sessions.

In terms of the way that information about the study was communicated, parents preferred face-to-face meetings and phone calls when being asked for informed consent for this intervention. Involving parents earlier in the process and giving more careful attention to how the information was delivered are strategies that may prove useful in the future [6]. Fletcher and Hunter propose methods such as use of teacher incentives to make sure that all students give their parents the consent forms; Research Assistants or other school personnel to follow up; colorful (neon colored) consent forms to attract attention of parents; and multiple waves for collecting consent [8]. Flory and Emmanuel - in reference to general and not specifically parental consent- suggest having neutral educators for one-to-one communication with study participants [40]. In line with suggestions from the literature, prior to embarking on recruitment, a tailored plan in collaboration with each of the schools principals can address the particularities of each school. In addition, as suggested above, partnering with school personnel – and particularly school heath personnel – can enhance the consent taking process. The proposed strategies in the literature are important components for a plan of action to adopt within participating schools.

In light of the perceived lack of understanding among parents of the value of research and the intricacies of protection of human participants, innovative strategies such as social marketing campaigns may be useful for raising awareness about the scope of waterpipe smoking among youth, and its consequences, as well as the importance and value of prevention research. Social marketing utilizes principles of marketing to promote behavior that is socially beneficial [41]. These campaigns could be followed by face-to-face meetings among parents and research teams as suggested by parents and could serve to further correct misconceptions and to highlight the importance of the planned intervention. In another youth intervention that took place in the Arab world, group meetings with parents, in which researchers explained the intervention and the process of participant selection, were shown to improve the rate of parental consent [20]. Spending more time with parents to explain the project and the consent process may be especially important in Qatar and other similar Arab countries, where experience with population-based research is recent and less familiar. Although the research team did suggest holding informational face-to-face meetings with parents to explain the study prior to requesting permission, schools system administrators - based on previous experience - thought it unlikely that parents would attend. Perhaps the idea would have greater appeal if sessions were offered in conjunction with other schools events, such as performances or parent teacher conference.

Parents in this study indicated that this was their first experience of being asked to give permission for their child’s participation in a research study. Principals stated that activities implemented within the school schedule are typically considered part of the education program, and therefore specific permission for participation is not requested. In the majority of schools who participated, principal’s approval and child’s assent were seen as sufficient for participation in activities. The lack of familiarity with the consent process required by research studies-- among both principals and parents-- may have contributed to the low rate of return of the permission forms.

The results also suggested a possible tendency for fathers to be more likely to give permission, and for parents to be more likely to give permission for girls’ participation over boys. Literature is equivocal on these issues. Parental gender was found to be a non-significant variable in active parental consent in a school-based research study in a mid-size city in the Midwest of the United States [42]. With regard to gender of the child, in research very similar to the study we describe here, Unger et al. [43] assessed parental permission for child (6th graders in California) participation in a tobacco prevention intervention. Similar to our results, they found that active parental consent procedures resulted in fewer boys participating (than passive consent procedures). Also, Jelsma et al. found that male students in South Africa were less likely to return consent forms [44]. This may be related to our finding that parents were less likely to consent to participation of boys. However, Secor-Turner et al. found no differences in permission rates by student gender [45]. This study, however, contrary to the others, included phone calls to parents who did not initially return consent forms – and thus social desirability bias may have influenced results.

### Limitations of the study

There are several limitations to this research. Overall, over 50% of consent forms were not returned. It is unclear if those parents ever received the forms, if children neglected to bring back completed forms, or if parents got the forms but chose not to return them because they were not interested in having their child participate (another way of not granting permission). We have no demographic or behavioral data that can compare the group that did return and did not return the consent form, and therefore cannot assess the extent to which we have a biased sample. The reasons for not giving permission of this latter group may be different than those listed above; and therefore, conclusions drawn about reasons for non-permission must be made only for parents who returned the consent forms. For parents who did not give consent, the request to explain their reasons was in free text format, rather than options that they could select from. This may explain the low response rate to this item. For the follow-up interviews, we were only able to access parents from 12 of the 40 schools. Other parents may have different reasons for not giving permission. In addition, only 28 parents agreed to be interviewed of 200 contacted. This may have resulted in a biased sample as evidenced by the high educational level of the cohort we reached. Also, perception of income compared to others was used as a proxy for SES, whereas school fees might have been a better proxy. For the 10% of parents who did not give permission based on the fact their child was not interested, we cannot verify if this was based on discussion with the child or an assumption made by the parents. Finally, the same field workers who led recruitment also carried out the interviews which could have biased interpretation of data. This concern is minimized by the concise, non-expansive responses provided by parents which limited opportunity for creative interpretation. Despite these limitations, this is the first study in the region to begin to document parental opinions of the informed consent process for a school-based intervention program.

## Conclusions

This study has implications for both practice and further research. Results of this research confirm that more attention and care needs to be given to the parental permission process. Researchers should enhance both the methods of communicating information to parents, and the specific information provided. As suggested in the literature and confirmed by our findings, direct face-to-face communication is recommended as one step in the informed consent process. Given the size of the expatriate population in Qatar, any direct communication should be conducted with parents in their native language as well as in the teaching language of the school. Also, permission forms need to be provided in English, and also in each of the respective languages of origin to which the school caters. Though time consuming, this ensures that the rights of participants are preserved and promoted and increases trust between researchers and parents. Moreover, in addition to the usual components of a permission form, attention and response to misperceptions and provision of detailed information regarding activities may help allay parental concerns and enhance the granting of permission for participation. In conclusion, we recommend that future research include additional formative research to better understand the parental permission process and the development of a plan of action to maximize informed parental permission. Finally, an important contribution to research in this area - that could also guide future practice - includes investigating the impact on parental permission of variables such as gender and educational level of the parent, gender of the child, language spoken at home, and type of school.

### Human subjects approval statement

The study protocol including all consent and assent procedures were approved by the Institutional Review Board at the Hamad Medical Corporation in Qatar.

## Electronic supplementary material

Additional file 1: Table S4: Main themes from the interviews and related quotes. (DOCX 16 KB)
