# Supplementary material for: Challenges to obtaining parental permission for child participation in a school-based waterpipe tobacco smoking prevention intervention in Qatar
Source: BMC Med Ethics. 2014 Sep 30;15:70. doi: 10.1186/1472-6939-15-70 (PMC4254406; doi:10.1186/1472-6939-15-70)
Supplement: Supplementary file 1 — Additional file 1: Table S4: Main themes from the interviews and related quotes. (DOCX 16 KB) [file 12910_2014_305_MOESM1_ESM.docx]

**Table S4 – Main themes from the interviews and related quotes**

| **Theme** | **Related Quotes** |
| --- | --- |
| **Locus of decision making for participation of children in activities** | "The choice to participate in any program is taken by my husband and myself first but my daughter has to agree also. So, the final decision is taken by my daughter when we both give approval for participation."  *Female, 31 to 35 years old, University level education* |
|  | "The final decision of whether my daughter should join in a project or not is mine, however I always asks for her opinion."  *Male, 40 years old, University level education* |
|  | "When it comes to deciding whether my daughter should join a study or not, I discuss it with my husband first and then we ask our daughter whether she would like to join."  *Female, 36 to 40 years old, Secondary level education* |
|  | "The decision of whether my son should join in a project or not is usually taken after a discussion with my son. If he doesn’t want to join I wouldn’t force him."  *Male, 58 years old, University* |
|  |  |
| **Content for the informed permission form** | "The consent was understandable, but maybe you need to add more details about the sessions that will be given to the students."  *Female, 42 years old, University level education* |
|  | "The confidentiality of students is the most important section in the consent."  *Female, 31 to 35 years old, Secondary level education*  "The procedures and risks part in the consent are important and the information is more than enough."  *Female, 36 to 40 years old, Secondary level education* |
|  | "I found the consent to be enough and thought my son would benefit a lot by joining."  *Female, 31 years old, Elementary level education* |
|  |  |
| **Communication with parents** | "I thinks that it’s better to let the parents know about programs through direct contact."  *Female, 31 to 35 years old, University level education* |
|  | "Even if you contacted the parents over the phone there are a lot who are strict and who will not agree to answer your questions except in one case which is parent meetings."  *Female, 48 years old, University level education* |
|  | "Parent meetings can be suggested as a way to reach parents, however it might not work since not all parents would be encouraged to attend. I think if meetings are short and planned at a time that is convenient for most parents then parents might be more encouraged to join."  *Male, 43 years old, elementary level education* |
|  | "The best way for reaching the parents is through sending a copy of the consent to their homes. It might be worth trying to have parents meetings although not all parents would come to such meetings. So maybe both should be done so that those parents who are busy can have a copy of the consent sent to them at home."  *Female, 42 years old, University level education* |
|  | "I think maybe if you have a general meeting with the parents it would be a good idea since you would be able to talk and explain more about the project."  *Male, 58 years old, University level education* |
|  | "I preferred if I was the one contacted directly over the phone rather than sending papers with my child about the study. This is because the child might take or might not take the paper home. The child might not know the importance of the topic and won’t give the paper to the parents."  *Male, 38 years old, High school level education* |
